# Alumina Concentration Detection Based on the Kernel Extreme Learning Machine

**DOI:** 10.3390/s17092002

**Published:** 2017-09-01

**Authors:** Sen Zhang, Tao Zhang, Yixin Yin, Wendong Xiao

**Affiliations:** 1 School of Automation & Electrical Engineering, University of Science and Technology Beijing, Beijing 100083, China; s20150646@ustb.edu.com (T.Z.); yyx@ies.ustb.edu.cn (Y.Y.); wdxiao@ustb.edu.cn (W.X.); 2 Key Laboratory of Knowledge Automation for Industrial Processes, Ministry of Education, Beijing 100083, China

**Keywords:** aluminum electrolysis, alumina concentration, extreme learning machine, kernel extreme learning machine, K-fold cross validation, predict

## Abstract

The concentration of alumina in the electrolyte is of great significance during the production of aluminum. The amount of the alumina concentration may lead to unbalanced material distribution and low production efficiency and affect the stability of the aluminum reduction cell and current efficiency. The existing methods cannot meet the needs for online measurement because industrial aluminum electrolysis has the characteristics of high temperature, strong magnetic field, coupled parameters, and high nonlinearity. Currently, there are no sensors or equipment that can detect the alumina concentration on line. Most companies acquire the alumina concentration from the electrolyte samples which are analyzed through an X-ray fluorescence spectrometer. To solve the problem, the paper proposes a soft sensing model based on a kernel extreme learning machine algorithm that takes the kernel function into the extreme learning machine. K-fold cross validation is used to estimate the generalization error. The proposed soft sensing algorithm can detect alumina concentration by the electrical signals such as voltages and currents of the anode rods. The predicted results show that the proposed approach can give more accurate estimations of alumina concentration with faster learning speed compared with the other methods such as the basic ELM, BP, and SVM.

## 1. Introduction

In recent years, with the rapid development of science and technology, many countries pay more and more attention to the enterprise’s energy conservation in the energy intensive area. 

The main target in the production process of aluminum electrolyte is to reasonably control the alumina concentration in the electrolytic cell to make the electrolytic cell operate at a small range of alumina concentrations so as to save the energy. In real production processes, it is a must to control the alumina concentration and keep it at a lower level, where there is no precipitation and no anodic effect to achieving a higher current efficiency and taking full advantage of electric power.

At present, many experts and scholars working on alumina concentration measurement focus on the soft measurement method that uses the indirect variables to measure the alumina concentration. This method not only reduces the manual analysis and improves the measurement precision of the alumina concentration, but it also provides a reliable guarantee for achieving advanced control.

Numerous investigations have been carried out on the soft sensing method for alumina concentration by the researchers [1]. A prediction model based on a wavelet neural network was proposed by Li et al. in [2]. A prediction method based on linear regression and orthogonal transform is applied to improve the accuracy of the alumina concentration forecast by Lin et al. in [3]. Yan and Liang proposed a predictive model of an aluminum reduction cell based on LS-SVM [4]. Li et al. [5] proposed a new fuzzy expert control method based on smart identification, multi-control mode, and decision making mechanisms to achieve alumina concentration prediction and real time control. The GM (1, 1) model is introduced into the aluminum concentration estimate by Zhang et al. [6]. However, the computational burden of the above nonlinear predictive models is still large when the dimension of the input variable increases. The learning speed and accuracy of these networks are, in general, far slower and cannot meet the requirements of real time detection.

The extreme learning machine (ELM) is a novel single hidden layer feed forward neural network proposed by Huang. In ELM, the input weights and the bias of hidden nodes are generated randomly without human tuning and the output weights are determined based on the method of least squares. Unlike the traditional feed forward neural network learning algorithm, ELM has fast training speed and gets rid of the opportunity to converge to local minima [7]. The salient features of ELM are that its hidden layer parameters do not require manual intervention and can be assigned randomly before training, and the output weight is determined analytically via the least squares estimation method, making it easy to implement with better generalization performance and faster learning speed [7,8,9]. Nowadays, because of its good generalization, the ELM algorithm has been applied in many aspects like image segmentation [8], fault diagnosis [9], human action recognition, and human computer interface [10], and so on. The initial weights of ELM were set randomly, so it made algorithm unstable. Huang and others proposed a KELM algorithm that takes the ideas of the kernel function to the extreme learning machine [1]. Zhou et al. [11] proposed Illumination correction of dyeing products based on Grey-Edge and kernel extreme learning machine. Zhang et al. [12] proposed a method for electricity price forecasting based on a kernel extreme learning machine. Compared with the ELM model, the KELM model has better stability and generalization abilities.

In this paper, we proposed a KELM based alumina concentration forecast model for the online detection. As the alumina concentration forecast field has little work on ELM or KELM based prediction models, our work is the first to tackle this problem with KELM. The experimental results showed that the proposed method has a better performance compared to the current approaches used in this area. 

The remaining parts of this paper are arranged as follows: Section 2 gives some preliminaries, including a brief introduction of ELM and KELM. The proposed KELM and alumina concentration prediction model is detailed in Section 3, including the model set up and the problem analysis of the proposed approach. The model for experimental implementation and evaluation is presented in Section 4. The discussions and conclusions are given in Section 5.

## 2. The Theory of the Extreme Learning Machine and the Kernel Extreme Learning Machine

### 2.1. Extreme Learning Machine

**Figure 1:**
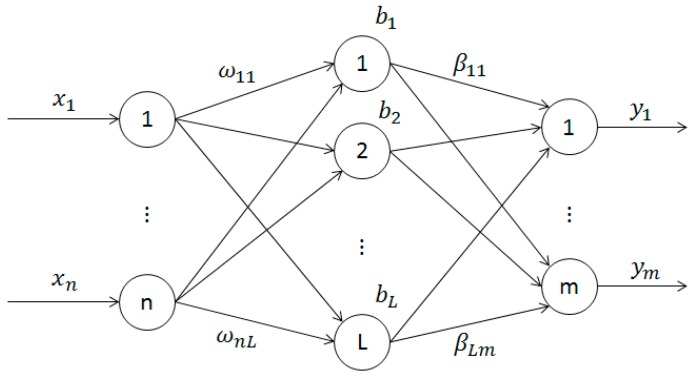
The network structure of the extreme learning machine model.

As a single hidden layer feed forward neural networks, the network structure of ELM model is shown in the Figure 1.

The input layer of the single hidden layer feed forward neural networks include n neurons which are correspond to the n input variables $x_{i}={\left[x_{i1},x_{i2},\ldots x_{in}\right]}^{T}$. The hidden layer includes L neurons, and the output layer includes m neurons which are correspond to the m output variables $y_{i}={\left[y_{i1},y_{i2},\ldots y_{im}\right]}^{T}$ [13]. Weight matrix w the input layer and the hidden layer of the network is shown in Equation (1).

(1) $$\omega ={\left[\begin{array}{cccc} \omega_{11} & \omega_{12} & \cdots & \omega_{1n}\\ \omega_{21} & \omega_{22} & \cdots & \omega_{2n}\\ \vdots & \vdots & \ddots & \vdots \\ \omega_{l1} & \omega_{l2} & \cdots & \omega_{\ln} \end{array}\right]}_{l\times n}$$

$w_{ji}$ is the input weight connecting the ith neuron of the input layer and the jth neuron of the hidden layer.

The bias of the hidden layer is $b_{i}={\left[b_{i1},b_{i2},\ldots b_{in}\right]}^{T}$. Connecting weight matrix $\beta_{i}={\left[\beta_{i1},\beta_{i2},\ldots \beta_{im}\right]}^{T}$ between the hidden layer and the output layer is shown in Equation (2).

(2) $$\beta =\left[\begin{array}{c} \beta_{1}^{T}\\ \beta_{2}^{T}\\ \vdots \\ \beta_{l}^{T} \end{array}\right]={\left[\begin{array}{cccc} \beta_{11} & \beta_{12} & \cdots & \beta_{1m}\\ \beta_{21} & \beta_{22} & \cdots & \beta_{2m}\\ \vdots & \vdots & \ddots & \vdots \\ \beta_{l1} & \beta_{l2} & \cdots & \beta_{lm} \end{array}\right]}_{l\times m}$$

$\beta_{ji}$ is the output weight connecting the jth neuron of hidden layer and the ith neuron of output layer. Suppose that the activation function of the hidden layer is *g*(*x*), the output of *Y* network is shown in Equation (3).

(3) $$Y={\left[y_{1},y_{2},\cdots y_{n}\right]}_{m\times n}$$

$$y_{j}={\left[\begin{array}{c} y_{1j}\\ y_{2j}\\ \vdots \\ y_{mj} \end{array}\right]}_{m\times 1}={\left[\begin{array}{c} \sum_{i=1}^{l}\beta_{i1}\left(\omega_{i}x_{j}+b_{i}\right)\\ \sum_{i=1}^{l}\beta_{i2}\left(\omega_{i}x_{j}+b_{i}\right)\\ \vdots \\ \sum_{i=1}^{l}\beta_{im}\left(\omega_{i}x_{j}+b_{i}\right) \end{array}\right]}_{m\times 1}$$

The above formula can be abbreviated as Equation (4).

(4) Hβ=Y

*H* is the output matrix of the hidden layer, and the ith column of *H* is the output of the ith hidden layer neuron corresponding to the input $x_{j}$, *H* is shown in Equation (5).

(5) $$H=\left[\begin{array}{cccc} g(w_{1}\cdot x_{1}+b_{1}) & g(w_{2}\cdot x_{1}+b_{2}) & \cdots & g(w_{l}\cdot x_{1}+b_{l})\\ g(w_{1}\cdot x_{2}+b_{1}) & g(w_{2}\cdot x_{2}+b_{2}) & \cdots & g(w_{l}\cdot x_{2}+b_{l})\\ \vdots & \vdots & \ddots & \vdots \\ g(w_{1}\cdot x_{n}+b_{1}) & g(w_{2}\cdot x_{n}+b_{2}) & \cdots & g(w_{l}\cdot x_{n}+b_{l}) \end{array}\right]$$

Compared with the traditional neural network, the input function approximation theory of the ELM algorithm needs to adjust the weights and the bias value [14,15]. The input weight and the deviation of the hidden nodes is generated randomly. So $\overset{\wedge }{\beta }$ is calculated by Equation (6). $\overset{\wedge }{\beta }$ is shown in Equation (7).

(6) $$\left\|H\overset{\wedge }{\beta }=Y\right\|=\min_{\beta }\left\|H\overset{\wedge }{\beta }=Y\right\|$$

(7) $$\overset{\wedge }{\beta }=H^{*}Y$$

$H^{*}$ is the generalized inverse matrix of *H*.

Approximating function of ELM algorithm is shown in Equation (8).

(8) $$f(x_{p})=h(x_{p})H^{T}{\left(\frac{1}{c}+HH^{T}\right)}^{-1}Y$$

### 2.2. Kernel Extreme Learning Machine

The initial weights of the ELM were set randomly, so it made algorithm unstable. Huang and others propose a KELM algorithm that takes the ideas of the kernel function to the extreme learning machine [1]. Compared with the ELM model, the KELM model has better stability and generalization ability. According to the theory of kernel function, specific form of the activation function which belongs to the hidden layer is unnecessary in the KELM model [16,17]. The inner product of matrix can be replaced by the kernel function which meets the Mercer theorem [18].

When the ELM uses the least squares solution of a linear function Hβ=Y, $HH^{T}$ is not a nonsingular matrix in the generalized inverse matrix $H^{*}=H^{T}{\left(HH^{T}\right)}^{-1}$ because of multicollinearity. This affects the prediction effect of the model. To avoid this problem, Huang introduces a parameter *C* into a diagonal matrix so that the eigen-values of the matrix are not zero, and the weight vector $\overset{\wedge }{\beta }$ is obtained by Equation (9) [10].

(9) $$\overset{\wedge }{\beta }=H^{T}{\left(\frac{1}{c}+HH^{T}\right)}^{-1}Y$$

The output expression can be expressed as

(10) $$f(x)=g(x)\beta =g(x)H^{T}{\left(\frac{1}{c}+HH^{T}\right)}^{-1}Y$$

The output matrix of the hidden layer can be expressed in Equation (13). $x_{i}={\left[x_{i1},x_{i2},\ldots x_{in}\right]}^{T}$ are *n* input samples, and *g*(*x*) is the output function of the hidden layer nodes. $HH^{T}$ can be expressed as

(11) $$H={\left[\begin{array}{c} g(x_{1})\\ g(x_{2})\\ \vdots \\ g(x_{n}) \end{array}\right]}_{n\times l}$$

(12) $$\begin{array}{l} HH^{T}={\left[\begin{array}{c} g(x_{1})\\ g(x_{2})\\ \vdots \\ g(x_{n}) \end{array}\right]}_{n\times L}*{\left[\begin{array}{c} g(x_{1})\\ g(x_{2})\\ \vdots \\ g(x_{n}) \end{array}\right]}_{n\times L}^{T}\\ ={\left[\begin{array}{ccc} g(x_{1})*g(x_{1}) & \cdots & g(x_{1})*g(x_{n})\\ \vdots & \ddots & \vdots \\ g(x_{n})*g(x_{1}) & \cdots & g(x_{n})*g(x_{n}) \end{array}\right]}_{n\times n} \end{array}$$

A kernel function satisfying the Mercer theorem is constructed to replace the inner product, it can be expressed as in Equation (13).

(13) $$HH^{T}\left(i,j\right)=K\left(x_{i},x_{j}\right)$$

Thus we can deduce the Equations (14) and (15).

(14) $$HH^{T}=\Omega_{ELM}=\left[\begin{array}{ccc} K(x_{1},x_{1}) & \cdots & K(x_{1},x_{j})\\ \vdots & \ddots & \vdots \\ K(x_{i},x_{1}) & \cdots & K(x_{i},x_{j}) \end{array}\right]=K(x_{i},x_{j})$$

(15) $$g(x)H^{T}={\left[\begin{array}{c} K(x,x_{1})\\ K(x,x_{2})\\ \vdots \\ K(x,x_{n}) \end{array}\right]}^{T}$$

Therefore, for a given training sample ($x_{i},y_{i}$), $x_{i}={\left[x_{i1},x_{i2},\ldots x_{in}\right]}^{T}$, $y_{i}={\left[y_{i1},y_{i2},\ldots y_{im}\right]}^{T}$. The output function of KELM algorithm is

(16) $$f(x)={\left[\begin{array}{c} K(x,x_{1})\\ K(x,x_{2})\\ \vdots \\ K(x,x_{n}) \end{array}\right]}^{T}{\left(\frac{1}{c}+\Omega_{ELM}\right)}^{-1}Y$$

In the model of learning machine based on kernel function, the value of the output function can be obtained by the particular form of the kernel function [19,20]. At the same time, the kernel function is used instead of the inner product of the matrix, so it is unnecessary to set the weight matrix w and the bias matrix of the hidden layer nodes b.

It can be seen that the kernel matrix is used instead of the random matrix in the algorithm to correct the random fluctuation caused by the random assignment in the previous algorithm and improve the accuracy and the generalization ability and stability.

The algorithm of the KELM can be summarized in the following step learning model. Given a training set, an activation function *g*(*x*) and the hidden neuron number l, we have the following steps.

Step 1 Assign kernel function and parameter *C*.
Step 2 Calculate the hidden layer output matrix *H*.
Step 3 Calculate the output weights $\overset{\wedge }{\beta }$.
Step 4 Calculate the output *f*(*x*).

## 3. Aluminum Concentration Detection in Aluminum Electrolysis Industry 

The paper collected experimental data from the field which include the current data of anode rods together with the voltage data between the anode rods and the cathode steel bars. In order to get the mathematical model between the input and output parameters, we have to obtain a certain amount of data samples to learn the weights and the structure of the neural network.

The data in this paper was collected from an aluminum electrolysis facility in Chongqing, China, and we chose a relatively stable electrolytic cell as the experimental cell in the work area. In this experiment, the data samples that correspond to the anode guide rod of two electrolytic cells were collected. 

**Figure 2:**
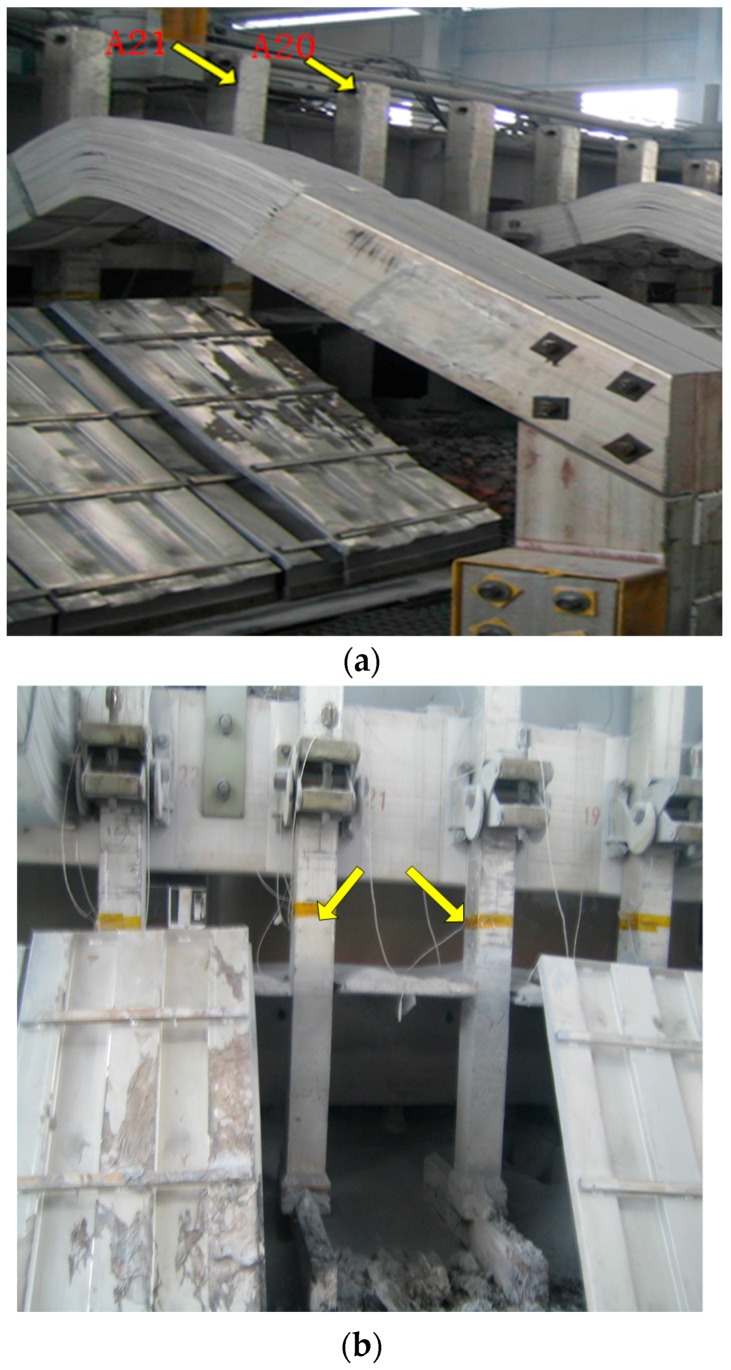
The experimental field. (**a**) The anode guide rod; (**b**) the point used to measure the voltage.

The current of the anode rods are from the A20 and A21 anode guide rod. Figure 2a shows the anode guide rod in the experiment. The method to measure the anode rod current is as follows: the anode rod is placed by a fixture with a voltage sensor and a temperature sensor, and we measure the voltage of the two points as shown by Figure 2b.

We calculate the resistance of the rod according to the size and the material of the section of the anode rod. We can calculate the anode rod current by using the voltage to divide the corrected resistor.

The rod resistance is calculated by Equation (17).

(17) R=a×(1+b×T)×L÷(W×H)

*R* is the rod resistance value of the demanded section; *T* is the real-time temperature of the rod; *a* and *b* are constant coefficients; *L*, *W*, and *H* are the length, width, and height of this section of the rod respectively; the rod current is calculated by Equation (18).

(18) $$I=\frac{U}{R}$$

*I* is the rod current; *U* is the voltage of the two measured points; *R* is the resistance of the two measured points.

Based on the above method, the whole measuring system consists in the anode rod isometric voltage drop measuring module, the anode rod temperature measuring module, STM32 microprocessor, RS-485 communication module, flash data storage module, and a reset and power module crystal oscillator circuit. 

The voltage signal between the anode guide rod and the cathode steel bar is collected by PCI1715U data acquisition card and the designed voltage protection circuit. In the field, the collected voltage data whose acquisition frequency is 10Hz is stored in the upper computer. The anode of the voltage is a wire that is drawn from the current acquisition device of the anode guide rod, and the cathode of the voltage is a wire that is drawn from the cathode steel bar under the experimental guide bar.

**Figure 3:**
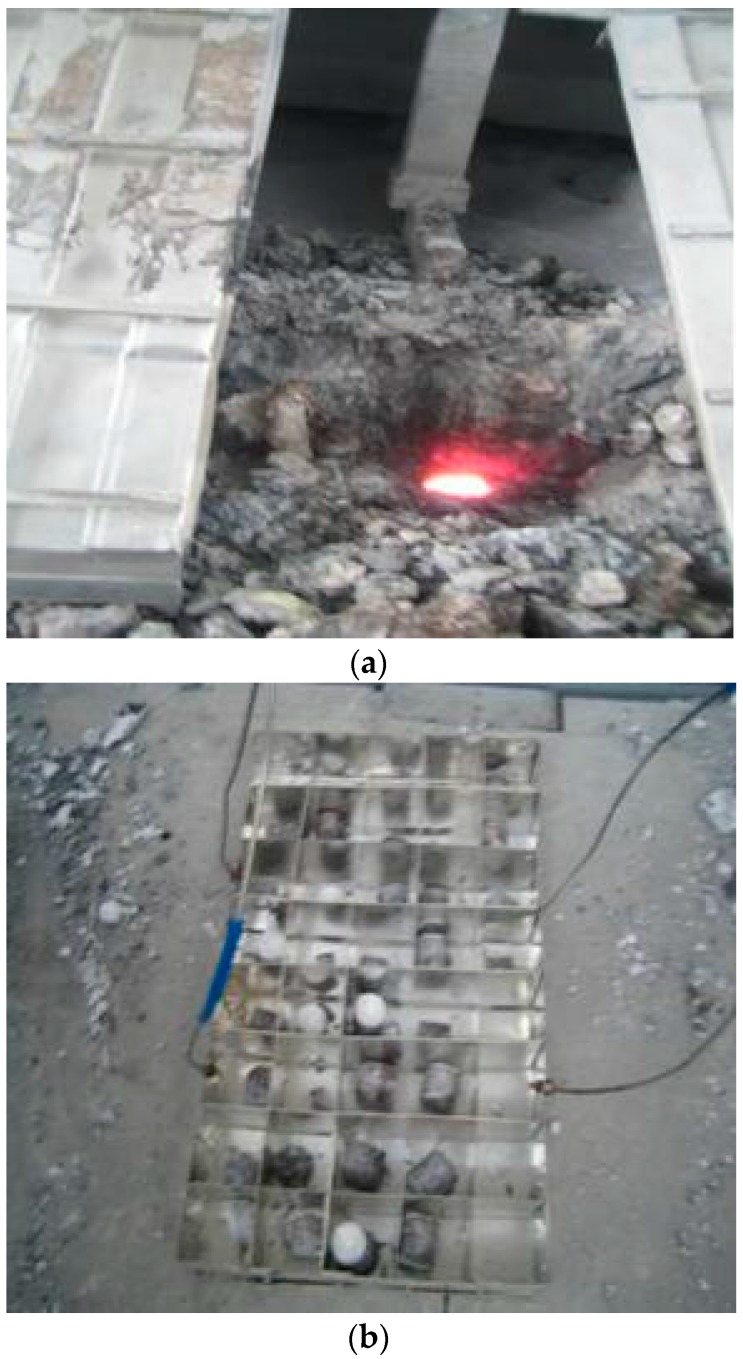
Field of collecting alumina concentration data. (**a**) The location of the electrolyte sampling; (**b**) The collected electrolyte samples.

At present, the alumina concentration in the electrolyte cannot be directly obtained, and the electrolyte sampling in the electrolytic cell can only be done manually by the spectral analysis equipment. Spectral analysis was carried out on the electrolyte samples to get the alumina concentration parameter. At the same time, we recorded the sampling time which is corresponded to the voltage and the current data in tine. The training samples and testing samples of the neural network algorithm are formed. Figure 3a shows the location of the electrolyte sampling. Figure 3b shows the collected electrolyte samples.

At the experimental site, it is a big challenge to improve the frequency of the sampling of the alumina concentration. It takes a lot of time to collect electrolyte samples, and it is also a complicated process to measure the alumina concentration with spectral analysis equipment. As a result of this limitation, the data collection team went to the factory to collect data more than 30 times. 

The principle of the algorithm is to establish the soft sensor model, then we set different parameters repeatedly for training, gradually narrowing the scope of optimum parameters by comparing all the experimental results, which involves the application of cross validation. The error of the model can be calculated while the best parameters are found. The averages of the multiple errors are the decisive factors to compare the effect of the soft sensor models. The detailed model set up is in Section 4. 

## 4. Experimental Results

### 4.1. The Process of Measuring Alumina Concentration Parameters in the Industrial Field

The current data of the anode guide rod, as well as the bipolar voltage data and the corresponding alumina concentration data, were collected at the same time. The training samples are input into the KELM model for learning to establish prediction model of alumina concentration. The test samples are input into the trained model to predict and analyze the results.

The specific experimental procedures are as follows:

Step 1 Measure the current data of the anode guide rod. (The current group of the project provides real-time current data.)
Step 2 Measure the voltage data between the anode rods and the cathode steel bars.
Step 3 Sampling the electrolyte solution under the experimental anode guide rod, and use the alumina concentration analyzer of the aluminum factory to obtain the corresponding alumina concentration in the laboratory analysis room.
Step 4 The KELM model of alumina concentration is established by training the current data of the anode guide rod, the voltage data between the anode rods and the cathode steel bars, and the alumina concentration.
Step 5 Adopting cross validation to comparing the alumina concentrations that are obtained by the alumina concentration analyzer with the alumina concentration are obtained by the KELM model in different parameters and measures their errors. The parameter of the model whose error is lower is the best parameter.
Step 6 The prediction error of BP model, LS-SVM model, and ELM model are compared, and the good prediction ability and robustness of the KELM model are verified.

### 4.2. K-Fold Cross Validation

The determination of model parameters is usually by minimizing generalization error estimate, namely taking the generalization error estimates as the objective function of the determination of model parameters. Generalization error refers to the model’s error indicators of the predicted value and actual value of independent inspection data, generally described by the mean square of prediction error [21].

K-fold cross validation is a method to estimate generalization error. The initial sample is split into *K* subsets that all have equal size. A subset is used to verify the model, and others subsets are used for training. The root-mean-square error indicator of K-CV is

(19) $$E=\frac{\left(E_{1}+E_{2}+\cdots +E_{K}\right)}{K}$$

(20) $$E_{i}=\sqrt{\frac{\sum_{j=1}^{n}{\left(y_{j}-\overset{\wedge }{y_{j}}\right)}^{2}}{n}},i=1,2,\cdots K$$

$E_{K}$ is the root-mean-square error that the Kth subset is used to test; *E* is the root-mean-square error of model; *n* is the number of test sets samples; $y_{j}$ is the actual value corresponding to the test set sample; $\overset{\wedge }{y_{j}}$ is the output value of the test set sample.

In this paper, the initial sample is split into five subsets whose size are all 30. Taking one of the subsets as the test set and the others as the training sets. Finally, the each subset is used to test. Each error is averaged. The model parameters that correspond to the minimum error are the best parameters.

### 4.3. The Experimental Results

#### 4.3.1. The Experimental Details of the Alumina Concentration Model Based on KELM

KELM model is set up to predict the alumina concentration that is non-stationary. The current data of the anode guide rod as well as the bipolar voltage data and the corresponding alumina concentration data at the same time were collected. At the same time, the sample set is divided into five subsets whose size are all 30. Taking one of the subsets as the test set and the others as the training sets. The training sets is used to train the prediction model and the testing sets is used to test the effect of the model. The best parameters, including the regularization parameter and the kernel parameter of KELM prediction model, are selected by cross validation. The test sets are input into the trained model to predict and analyze the results. The experimental data indicate that KELM algorithm has characteristics of higher speed and greater accuracy when compared with the BP algorithm, LSSVM algorithm, and ELM algorithm. The proposed algorithm provides a feasible scheme in practical application for the online measurement of alumina concentration in the work site.

In the experiment, the sampling frequency of the collecting device that obtained the anode voltage data and the anode rod current data is 10 Hz, that is to say it can only get 10 sets of data within one second. However, the electrolyte samples in an electrolytic cell can only collect a sample within one second. Thus, in order to get training and testing samples of the model, the voltage and the current must be averaged.

**Figure 4:**
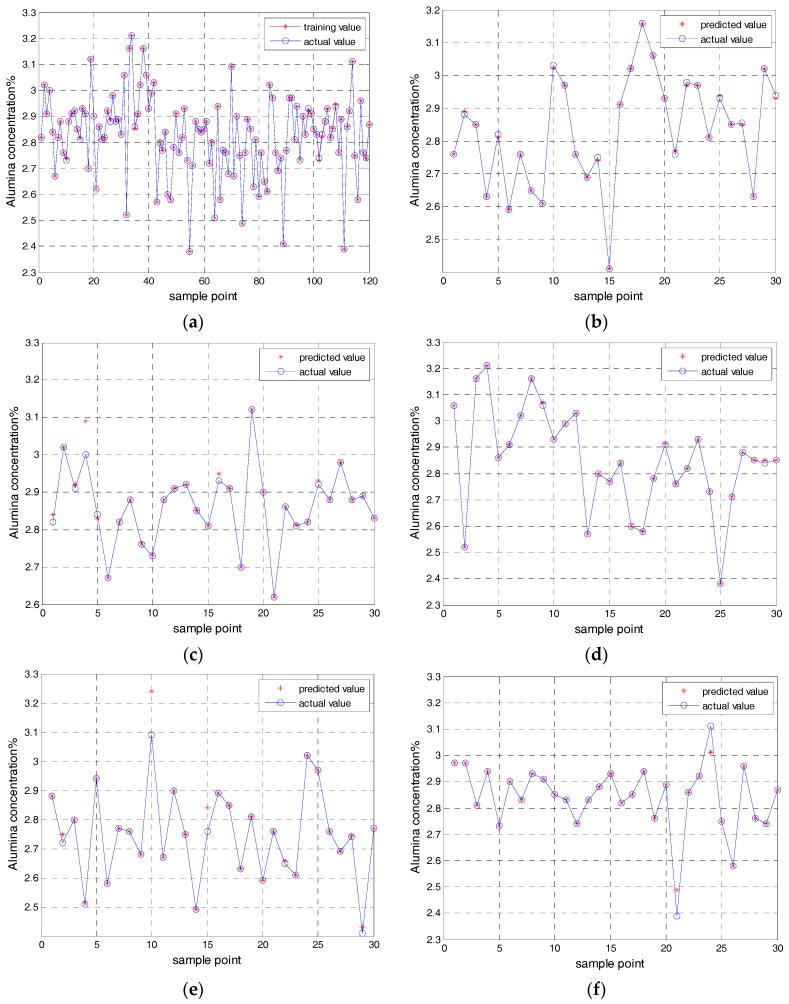
The training value and the predicted value of the KELM soft sensor model. (**a**) The training value of the first test subset; (**b**) The predicted value of the first test subset; (**c**) The predicted value of the second test subset; (**d**) The predicted value of the third test subset; (**e**) The predicted value of the fourth test subset; (**f**) The predicted value of the fifth test subset.

Because of the limited time for collecting data in the field, and the limitations of various conditions in the industry, 150 sets of training and test sample data were obtained after preliminary analysis and pretreatment of the A20 anode guide rod. A total 150 sets of samples are split into 5 subsets whose size are all 30. Taking one of the subsets as the test set and the others as the training sets. In other words, 120 sets of training samples are used to train the prediction model. 30 sets of test samples are used to validate the effect for the prediction model. The actual value of 30 sets of data is compared with the predicted value. Finally, each subset is used to test, and obtains a corresponding error. Figure 4a shows the training value of the alumina concentration model based on the KELM in the first test subset. Figure 4b shows the predicted value of the alumina concentration model based on the KELM in the first test subset. Figure 4c shows the predicted value of the second test subset of the alumina concentration model based on the KELM. Figure 4d shows the predicted value of the third test subset of the alumina concentration model based on the KELM. Figure 4e shows the predicted value of the fourth test subset of the alumina concentration model based on the KELM. Figure 4f shows the predicted value of the fifth test subset of the alumina concentration model based on the KELM.

Through the application of the cross validation and the KELM model, five root-mean-square error (RMSE) were obtained by five test subsets. The average of the five root-mean-square errors are used as a standard to measure the effect of the model. Five root-mean-square errors (RMSE) of the KELM model are shown in Table 1.

The average of the five root-mean-square error is used as a standard to measure the effect of the model. The RMSE of the alumina concentration model based on the KELM is

(21) $$E=\frac{\left(E_{1}+E_{2}+\cdots +E_{K}\right)}{K}=0.00528321$$

#### 4.3.2. The Experimental Result Comparison with BP, LSSVM, and ELM

To test and verify the prediction effect of KELM model, the same sample set is input respectively into the BP model, the LSSVM model and the ELM model to do the experiments. 

**Figure 5:**
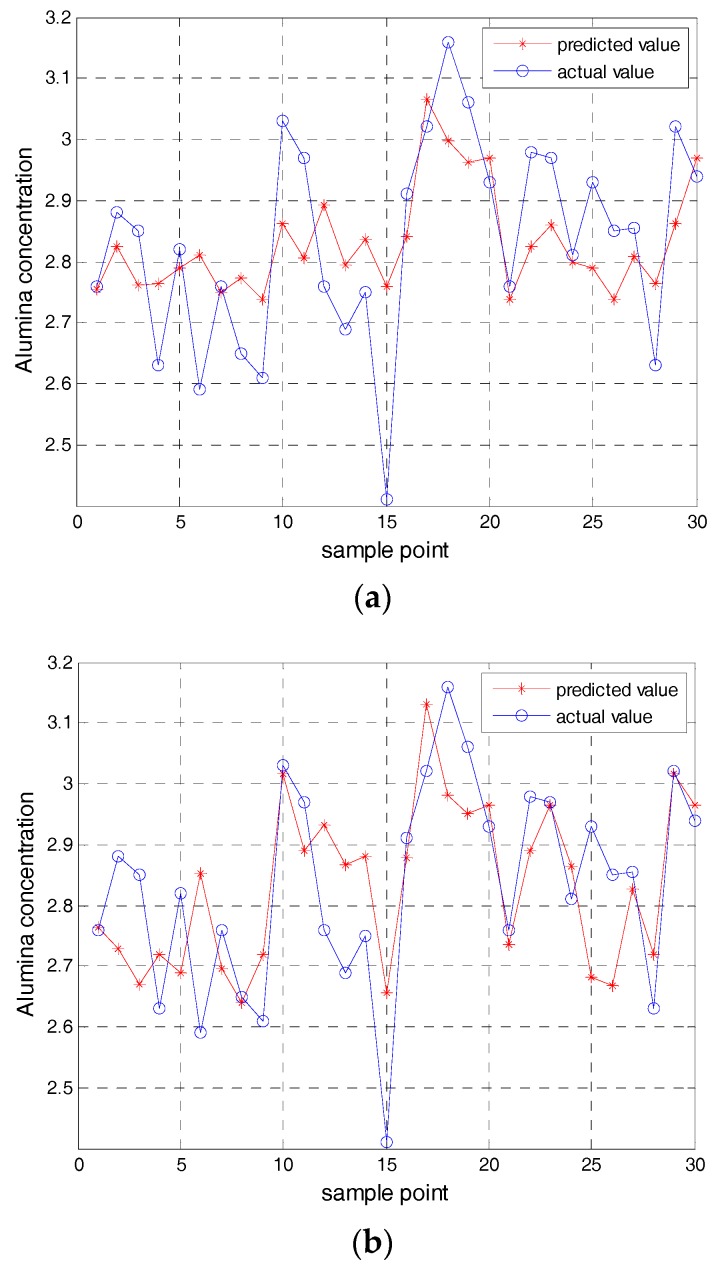

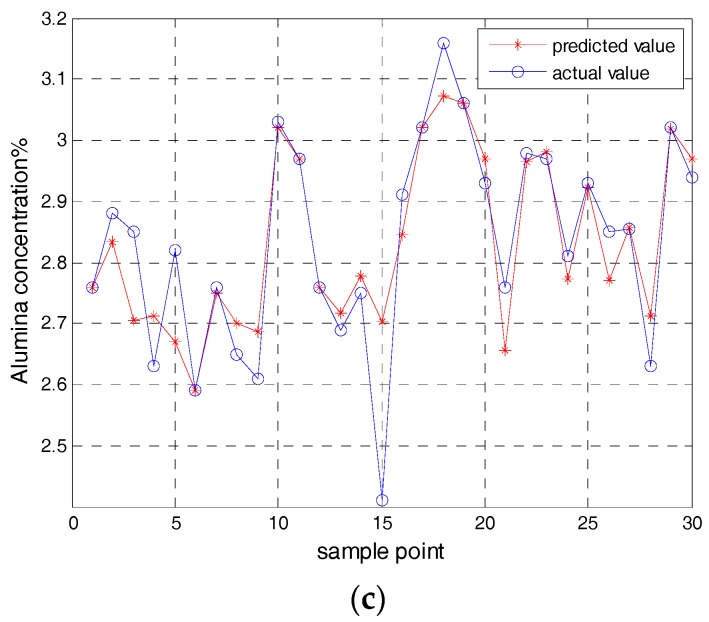
The predicted results of the other models. (**a**) The result of the BP model; (**b**) The result of the ELM model; (**c**) The result of the ELM model.

At the same time, the prediction results of each model are analyzed and compared, and the prediction results are shown in Figure 5. Figure 5a shows the predicted value of BP model in the first test subset. Figure 5b shows the predicted value of LS-SVM model in the first test subset. Figure 5c shows the predicted value of the ELM model in the first test subset.

### 4.4. Discussion of the Alumina Concentration Model

Each model has different parameter settings, and the list of parameters for each model is shown in Table 2. 

From the above figures, the BP model, the LSSVM model, and the ELM model are not good enough while the training and the predicted results of the KELM model are better. Comparing the training time, testing time, and root-mean-square error of various models under the same sample, and comparing results as shown in Table 3. As can be seen from Table 3, the training time, the test time, and the root mean square error of the KELM model are not only minimal, but also the predicted effect of the KELM model is better than that of the BP model, the LSSVM model, and the ELM model. In general, the KELM model has an obvious advantage over other models.

## 5. Conclusions

In this paper, the KELM soft sensor model is built to predict the alumina concentration. The proposed KELM has better generalization performance than BP, LSSVM, and ELM in most cases. The traditional classic gradient-based learning algorithms may face several issues like local minima, improper learning rate, and over fitting, etc. The KELM tends to reach the solutions in a straightforward manner without such trivial issues. 

In this paper, in order to verify that the proposed approach has better performance than the other approaches, several performance criteria were applied to evaluate the algorithms. The root-mean-square error (RMSE) and the mean absolute error together with the K-fold cross validation were selected to evaluate the accuracy of the prediction model. The training time and testing times were used to evaluate the training speed and the testing speed. As can be seen from Table 3, the training time, the testing time, the mean absolute error, and the root mean square error of the KELM model are smaller than those of the BP model, the LSSVM model, and the ELM model. This shows that the KELM model has obvious advantages over the above models.

## Figures and Tables

**Table 1:** Five root-mean-square error (RMSE) of KELM model.

Test Subsets	Root-Mean-Square Error (RMSE)
The first test subset	0.00431167
The second test subset	0.00629538
The third test subset	0.00340161
The fourth test subset	0.00492386
The fifth test subset	0.00748353

**Table 2:** The list of parameters for each model.

	BP Model	LSSVM Model	ELM Model	KELM Model
Number of input samples	2	2	2	2
Number of output samples	1	1	1	1
Number of hidden layer nodes	100	/	100	/
kernel function	/	RBF	/	RBF
Kernel parameter	/	1	/	1
Regular parameter	/	20	/	20
Activation function	Sigmoid	/	Sigmoid	/

**Table 3:** The comparison of the training time, the testing time, and the root mean square errors.

	RMSE	Training Time/s	Testing Time/s
BP	0.12704836	1.172490391	0.020361037
LSSVM	0.1269727	0.446370848	0.022538922
ELM	0.11958055	0.315678179	0.011760205
KELM	0.00528321	0.231460549	0.006411933
